# Long-Term Fertilisation Regimes, Crop Yield and Rhizosphere Microecology in a Wheat–Soybean Rotation System

**DOI:** 10.3390/plants15193034

**Published:** 2026-10-04

**Authors:** Jingyang Li, Qiuju Wang, Xinyu Li, Zhuo Zhang, Yanxia Liu, Qingying Meng, Jiahe Zou, Xin Liu, Yifei Luo, Zhenhua Guo

**Affiliations:** 1 Key Laboratory of Black Soil Protection and Utilization, Ministry of Agriculture and Rural Affairs, Heilongjiang Academy of Agricultural Sciences, Harbin 150086, China; 2 Heihe Multidisciplinary Observation and Research Station, Ministry of Agriculture and Rural Affairs, Heihe 164300, China; 3 Animal Husbandry Research Institute, Heilongjiang Academy of Agricultural Sciences, Harbin 150086, China

**Keywords:** ground vegetation, long-term, molecular docking, root, scanning electron microscopy

## Abstract

Long-term fertilisation must sustain crop yield and rhizosphere microbial functions in wheat–soybean rotations, but interannual responses remain unclear. We examined 12 treatments in a long-term field experiment established in 1979 and continuing through 2025 in Heilongjiang, China; three within-plot soil composites per treatment represented the 2025 microbiome snapshot. Long-term wheat and soybean grain and straw yield, rhizosphere and non-rhizosphere metagenomes, bacterial diversity, functional gene profiles, taxonomic composition, yield–microbe correlations, and structural equation modelling were assessed. The displayed wheat grain yields differed among treatments, with the median approaching 2500 kg ha^−1^ under manure plus medium-rate mineral fertiliser. Soybean grain yield exceeded 2000 kg ha^−1^ under manure plus low- or medium-rate mineral fertiliser and was higher than under high-rate mineral fertiliser alone. Separate exploratory treatment-only PERMANOVAs returned R^2^ values of 0.7578 and 0.8227 for rhizosphere and non-rhizosphere functional profiles, respectively; the profiles were within-plot subsamples. *Mesorhizobium*, *Lysobacter*, *Gemmatirosa*, and *Pseudolabrys* were positively correlated with crop yield. Structural equation modelling linked available phosphorus to wheat yield and rhizosphere diversity and linked available potassium to soybean yield. These descriptive findings suggest crop-specific fertilisation patterns and possible associations of yield with soil nutrients and rhizosphere microbiota, indicating that productivity and microbial responses should inform long-term nutrient management.

## 1. Background

The wheat (*Triticum aestivum* L.)–soybean (*Glycine max*) rotation system is an important agricultural production system worldwide that plays a critical role in maintaining food security and promoting sustainable agriculture [1,2]. Rational fertilisation in wheat–soybean rotation systems has long been regarded as an effective agronomic practice for increasing crop yield [3,4]. Although long-term chemical fertiliser application alone can increase crop biomass in a given season, it can also induce ecological degradation problems, including soil acidification, soil microecological balance disruption, and reduced nutrient-use efficiency [5,6,7,8]. In contrast, appropriate combined organic and chemical fertiliser application has been shown to increase soil organic carbon, improve the root-zone microenvironment, and restructure microbial communities [6,9].

The rhizosphere is the key microsite in which plant roots exchange substances and communicate with the surrounding soil, and its microecosystem stability determines crop nutrient acquisition and plant health [10]. Rhizosphere microbial communities undergo spatial and temporal succession under different fertilisation regimes [11,12]. Beneficial bacterial genera can promote crop growth and suppress soil-borne diseases through mechanisms such as symbiotic nitrogen fixation in root nodules, phosphate solubilisation, and antimicrobial metabolite secretion [13,14,15].

Based on a 46-year long-term wheat–soybean rotation field experiment, this study systematically evaluated the effects of 12 fertilisation treatments on soil ecological development. The objective was to examine associations among long-term fertilisation, crop yield, and the rhizosphere microbial community, providing a theoretical basis for long-term management strategies that simultaneously maintain a high and stable yield and preserve soil microecological health.

## 2. Results

### 2.1. Effects of Long-Term Fertilisation Regimes on Crop Yield

**Figure 1:**
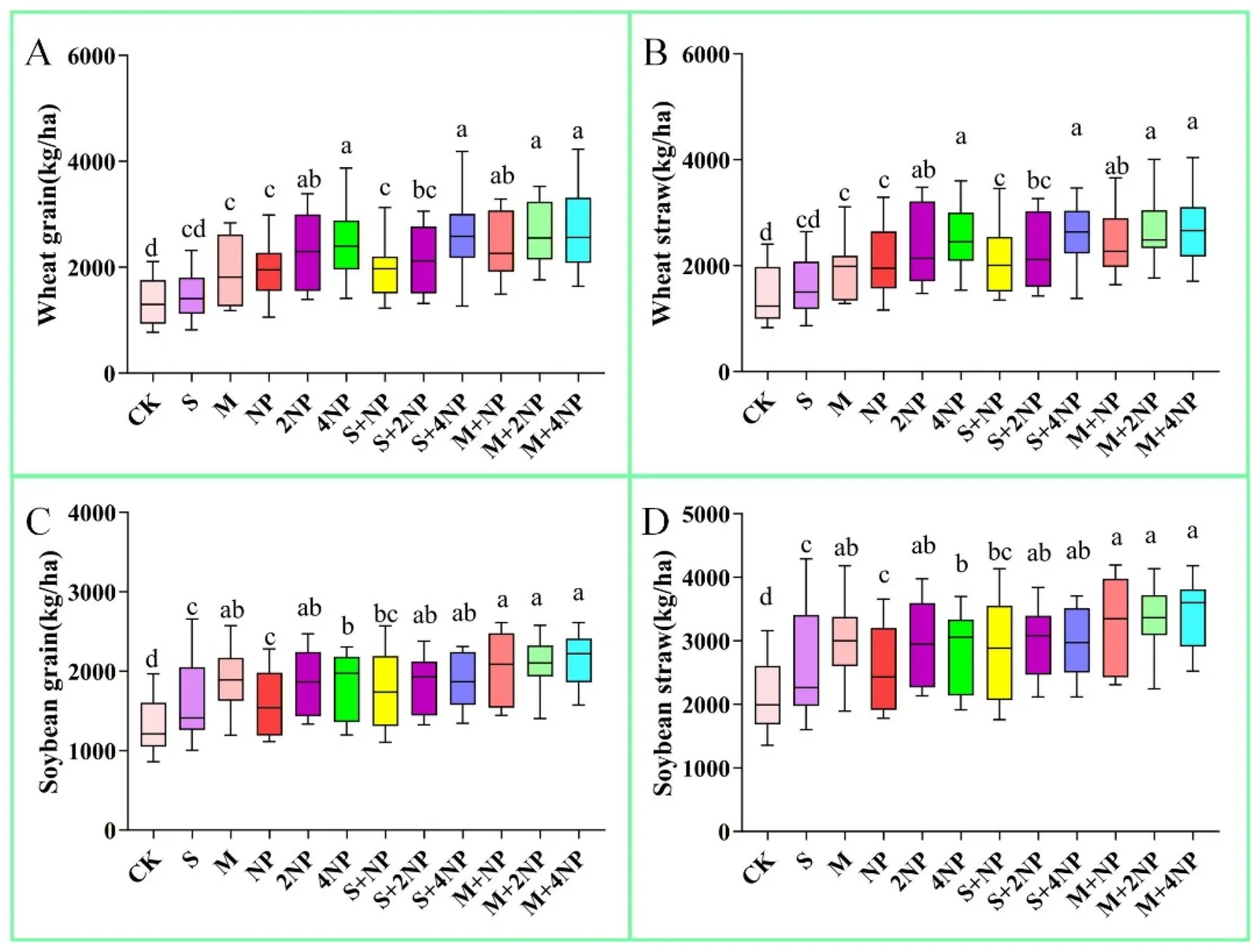
Long-term effects of fertilisation treatments on crop yield (1979–2025). (**A**,**B**) Wheat grain and straw yield, respectively. (**C**,**D**) Soybean grain and straw yield, respectively. Different lowercase letters above the boxplots indicate significant differences among treatments (*p* < 0.05).

The displayed long-term yield records varied among treatments. Wheat grain and straw yield was lowest in CK and S (Figure 1A,B). Wheat grain and straw yield increased with increasing chemical fertiliser input. M + 4NP, M + 2NP, and S + 4NP treatments showed the greatest increases in wheat yield. The median wheat grain yield under M + 2NP approached 2500 kg ha^−1^.

Soybean biomass responded to fertilisation in a pattern different from that of wheat. Although 4NP maintained a relatively high wheat yield, soybean grain and straw yield was lower than that under treatments containing organic manure (M, M + NP, M + 2NP, and M + 4NP). The median soybean grain yield exceeded 2000 kg ha^−1^ under M + NP and M + 2NP and was significantly higher than that under 4NP and S + 4NP (Figure 1C,D), indicating that long-term organic manure input is critical for maintaining high soybean productivity.

**Figure 2:**
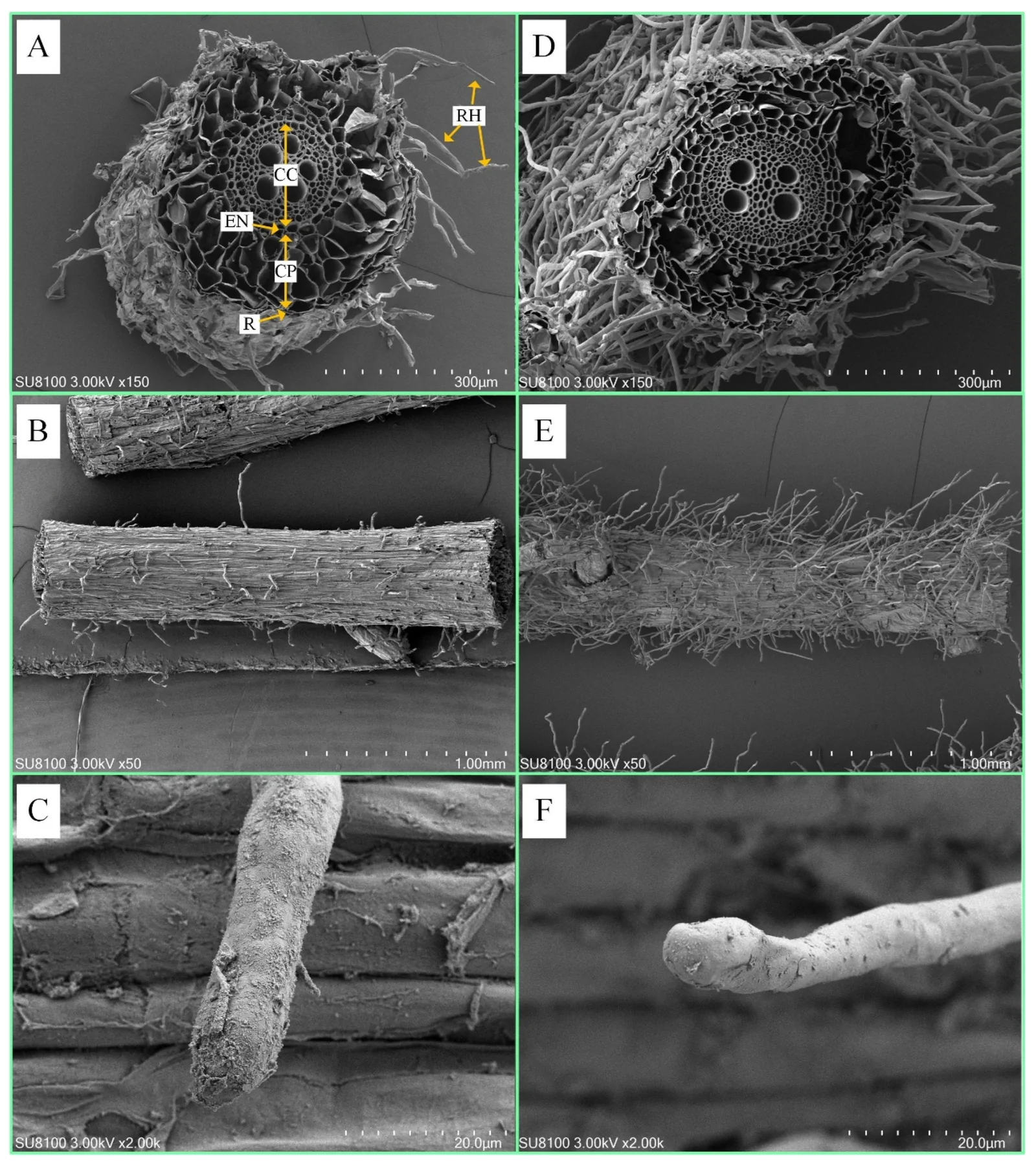
Scanning electron microscopy images of wheat root morphology. (**A**–**C**) Root cross-sections, epidermal cells, and root hair characteristics under the unfertilised control (CK); (**D**–**F**) Root characteristics under organic manure combined with high-rate chemical fertiliser (M + 4NP). CC, central cylinder; EN, endodermis; CP, cortical parenchyma; R, rhizodermis; RH, root hair.

In representative scanning electron microscopy images, wheat roots under M + 4NP (Figure 2D–F) appeared to have more visible root hairs than those under CK (Figure 2A–C).

### 2.2. Alpha Diversity of Rhizosphere Bacterial Communities and the Effect of the Rhizosphere

**Figure 3:**
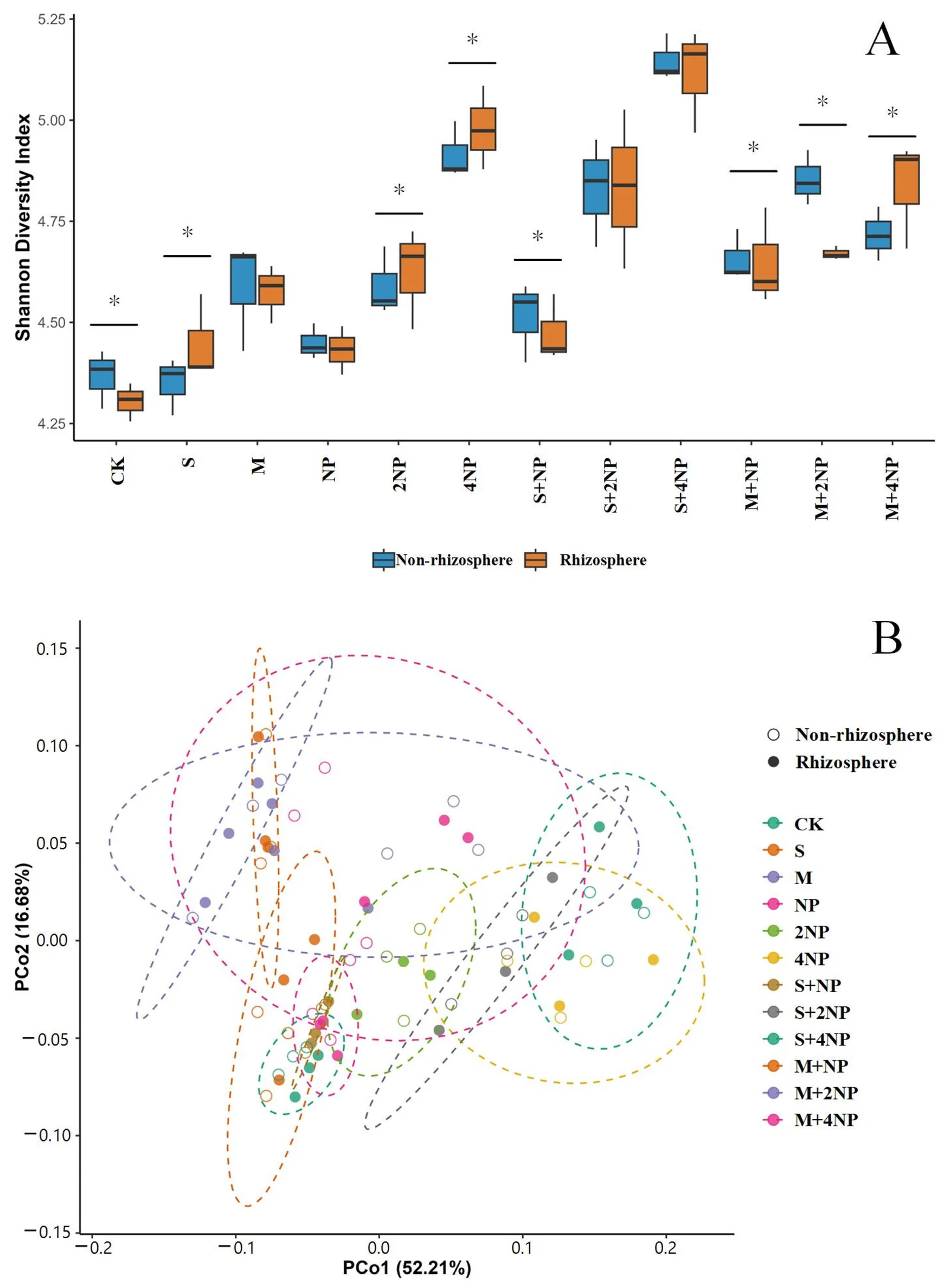
Alpha and beta diversity of rhizosphere and non-rhizosphere bacterial communities. (**A**) Paired comparison of bacterial Shannon diversity indices between rhizosphere (orange boxes) and non-rhizosphere (blue boxes) soils under 12 fertilization treatments (* indicates a significant difference between paired groups according to a paired *t*-test, *p* < 0.05). (**B**) Principal coordinate analysis (PCoA) of microbial functional gene composition based on a Bray–Curtis distance matrix.

Comparison of the microbial Shannon index between rhizosphere and non-rhizosphere soils (Figure 3A) demonstrated an effect of the rhizosphere. Under most fertilisation treatments, including CK, S, 4NP, and M + 2NP, the Shannon diversity was highly significantly different between non-rhizosphere and rhizosphere soils. For example, under 4NP, the mean Shannon index was 4.91 in non-rhizosphere soil and increased significantly to 4.98 in rhizosphere soil. Under S + 4NP, the Shannon diversity exceeded 5.10 in both rhizosphere and non-rhizosphere soils, indicating that the combination of straw and high-rate chemical fertiliser amplified and reshaped microbial diversity in the rhizosphere microenvironment.

### 2.3. Analysis of Microbial Functional Gene Abundance in Rhizosphere and Non-Rhizosphere Soils

Metagenomic functional gene abundance profiles for rhizosphere and non-rhizosphere soils were combined for PCoA ordination (Figure 3B). PCo1 and PCo2 explained 52.21% and 16.68% of the total variation, respectively, and the samples formed distinct dual clusters based on fertilisation treatment and spatial compartment (rhizosphere/non-rhizosphere). Separate treatment-only PERMANOVAs are reported in Table 1: for rhizosphere profiles, the original analysis returned R^2^ = 0.7578 (F = 6.83, *p* = 0.001), and for non-rhizosphere profiles, it returned R^2^ = 0.8227 (F = 10.12, *p* = 0.001). Each analysis contained 36 profiles (total df = 35), with three within-plot composites per treatment. Therefore, variation in the microbial functional profile of the wheat–soybean rotation system was primarily driven by the long-term fertilisation regime and characterised by pronounced rhizosphere compartment differentiation.

### 2.4. Dominant Rhizosphere Microorganisms

**Figure 4:**
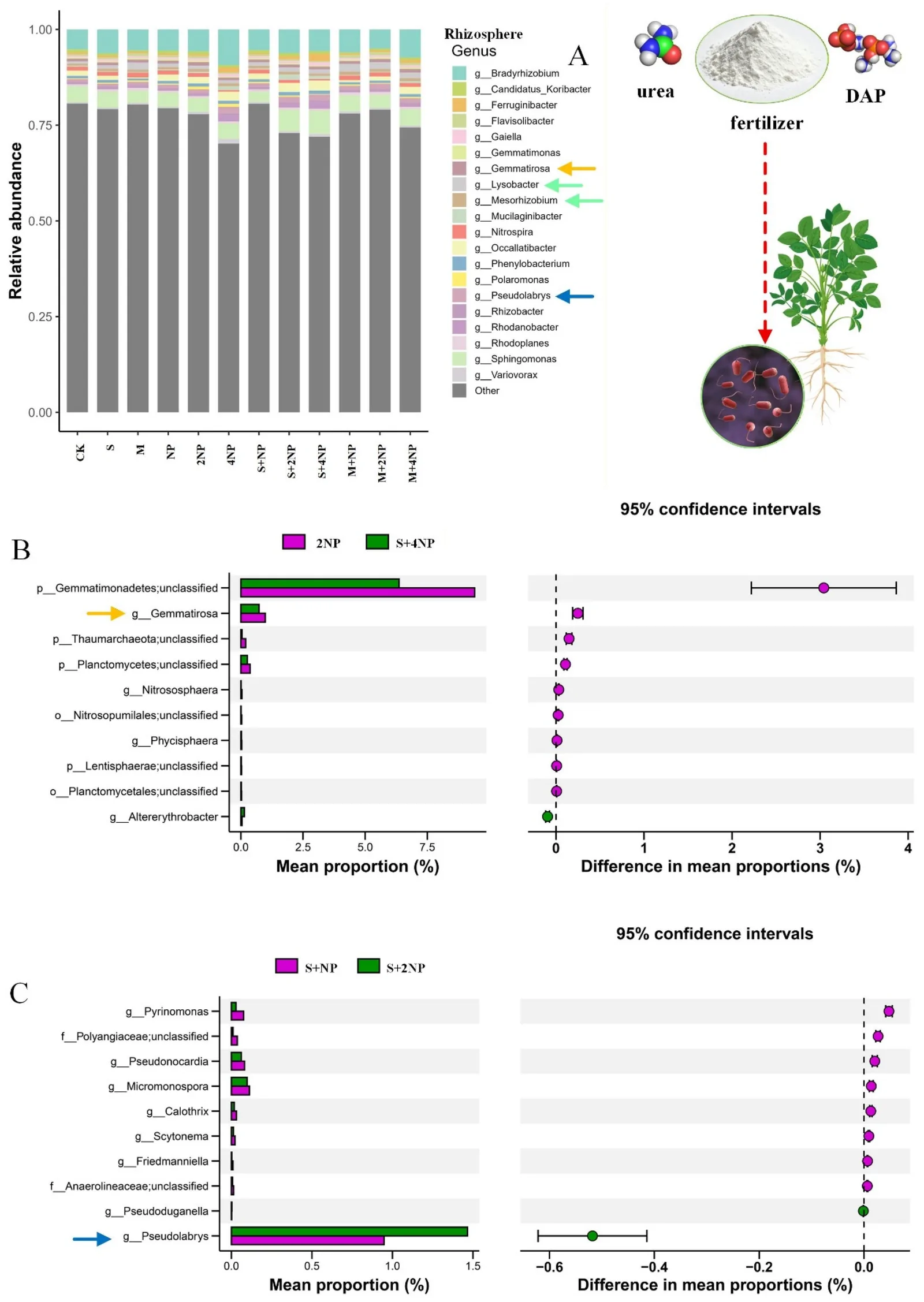
Taxonomic composition and differential enrichment of the rhizosphere microbiome. (**A**) Stacked bar plot of the relative abundance of the top 20 dominant genera under each fertilisation treatment. (**B**,**C**) Significantly different taxa identified using STAMP: (**B**) genus-level abundance between 2NP and S + 4NP; (**C**) genus-level abundance between S + NP and S + 2NP.

Based on rhizosphere soil metagenomic sequencing data, the relative abundance of the top 20 dominant microbial genera was calculated for each treatment (Figure 4A). Community composition was visualised using stacked bar plots, and means ± standard deviations were calculated to compare the effects of fertilisation treatments on the dominant microbial groups.

### 2.5. Functional Enrichment and Identification of Differential Taxa in the Rhizosphere Microecosystem

Taxonomic composition and differential abundance analyses showed that long-term fertilisation significantly altered the assembly of the core rhizosphere microbial community. LEfSe analysis (Supplementary Figure S1) revealed the treatment-specific enrichment of rhizosphere microorganisms. For example, *Acidobacteria* were significantly enriched under CK, and *Sphingomonas* was significantly enriched under S + 4NP.

Genus-level STAMP analysis (Figure 4B) showed that key genera, such as *Gemmatirosa*, were significantly enriched under 2NP compared to S + 4NP. *Pseudolabrys* and other key genera were significantly enriched under S + 2NP compared to S + NP (Figure 4C). Additional genus-level STAMP differential analyses are shown in Supplementary Figure S2.

The clustered heatmap of the top 50 enriched KO pathways (Supplementary Figure S3) showed that the two-component system was highly abundant under all treatments, with TPM values of approximately 25,000. Additionally, pyruvate metabolism, purine metabolism, ribosome-related functions, and ABC transporters showed high functional gene abundance in rhizosphere soils across all treatments.

### 2.6. Pearson Correlations Between Dominant Rhizosphere Genera and Crop Yield

**Figure 5:**
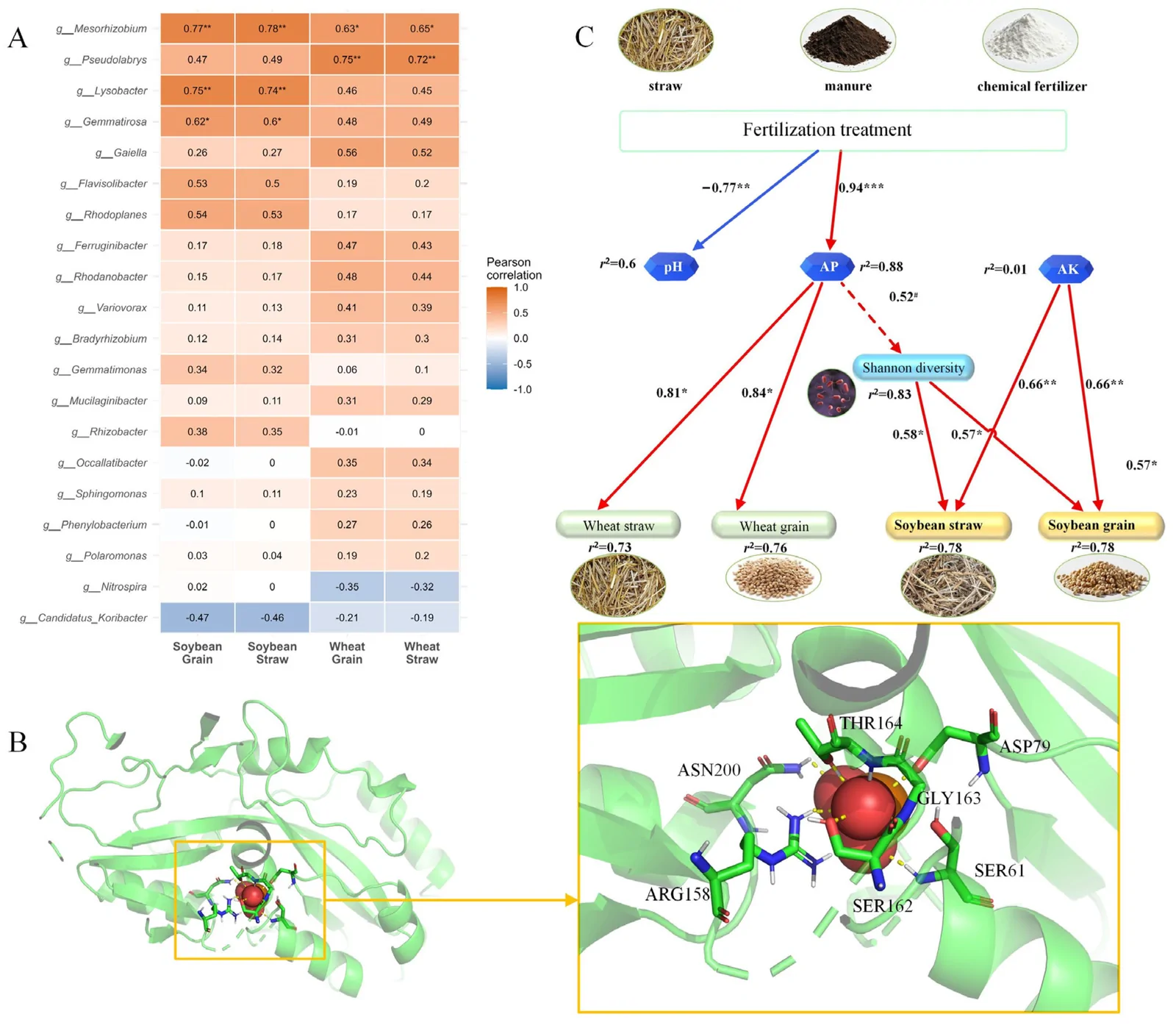
Correlation network and structural equation modelling of yield drivers. (**A**) Pearson correlation heatmap between the abundance of the top 20 dominant rhizosphere genera and wheat/soybean yield (grain and straw); red indicates positive correlations, and blue indicates negative correlations (* *p* < 0.05, ** *p* < 0.01). (**B**) Molecular docking of the *Lysobacter* PstS protein with phosphite; amino acid residues located within 5 Å of the phosphite molecule are labelled. (**C**) Structural equation model of cascading regulatory pathways in the fertilisation system. Solid lines indicate significant pathways; dashed lines indicate marginally significant pathways; numbers adjacent to the pathways are standardised path coefficients. *** *p* < 0.001, ** *p* < 0.01, * *p* < 0.05, # *p* < 0.1. Red arrows indicate positive effects, and blue arrows indicate negative effects.

The Pearson correlation heatmap (Figure 5A) identified core rhizosphere genera associated with crop productivity. *Mesorhizobium* was positively associated with soybean yield and showed positive correlations with soybean grain (*r* = 0.77) and straw yield (*r* = 0.78). *Lysobacter* similarly showed positive correlations with soybean grain (*r* = 0.75) and straw yield (*r* = 0.74). *Gemmatirosa* was also positively correlated with soybean yield. These results suggest that these genera are important microecological factors associated with the maintenance of soybean rhizosphere health and enhancement of nitrogen fixation-related soybean biomass.

*Pseudolabrys* was positively associated with wheat yield and showed positive correlations with wheat grain (*r* = 0.75) and straw yield (*r* = 0.72), suggesting a positive role in regulating nutrient transformation in the wheat rhizosphere. Figure 5B shows the molecular docking results for the *Lysobacter* PstS protein with phosphite, revealing a binding energy of −4.93 kcal/mol.

### 2.7. Structural Equation Model of Associations Among Soil Nutrients, Microbial Diversity, and Yield

Systematic path analysis using the structural equation model developed for the rotation production system (Figure 5C) showed that long-term fertilisation had a highly significant positive effect on available phosphorus (AP) (0.94 ***), which exerted strong positive effects on wheat grain (0.81 *) and straw yield (0.84 *). The fitted path linked fertilisation intensity to lower soil pH (−0.77). In the rhizosphere microecosystem, AP accumulation showed a marginally significant positive pathway to rhizosphere microbial diversity (Root Shannon) (0.52 †, R^2^ = 0.83). The fitted paths from rhizosphere microbial diversity to soybean straw and grain yield had coefficients of 0.58 and 0.57, respectively. The fitted paths from available potassium (AK) to soybean straw and grain yield had coefficients of 0.65 and 0.66, respectively.

## 3. Discussion

### 3.1. Differential Mechanisms by Which the Fertilisation Regime Regulates Crop Yield

Structural equation modelling identified associations among fertilisation regimes, measured soil properties, microbial diversity, and yield in the wheat–soybean rotation experiment. 4NP increased wheat grain and straw yield by increasing soil AP accumulation (Figure 5C). However, this yield-increasing strategy based solely on readily available mineral nutrients limited soybean production. Higher median soybean yield was observed in treatments containing organic manure, with the median yield under M + 2NP exceeding 2000 kg ha^−1^ (Figure 1C,D). Long-term organic manure application effectively buffered the decline in soil pH caused by chemical fertiliser and, in combination with AK, maintained a high Shannon diversity for rhizosphere bacteria.

### 3.2. Functional Assembly of the Core Rhizosphere Microecosystem and Yield Responses

In this study, decades of field management resulted in differences in the effects of the rhizosphere, and the original rhizosphere-only treatment PERMANOVA returned R^2^ = 0.7578 for functional gene composition (Table 1). The top 20 dominant genera included *Mesorhizobium*, *Lysobacter*, *Gemmatirosa*, and *Pseudolabrys* (Figure 4A), and all four genera were positively associated with crop yield (Figure 5A).

During evolution, soybean established a mutualistic symbiosis with *Mesorhizobium*, which is important for root nodule formation and promotes soybean growth [16,17]. In the core rhizosphere microbiome, *Mesorhizobium* showed a positive correlation with soybean biomass (*r* = 0.77) (Figure 5A). This supports the importance of *Mesorhizobium* in legume symbiosis, as it successfully infects roots under complex soil competition, forms nodules, and continuously provides soybean with essential biological nitrogen fixation [18,19].

Machine-learning models and soil biomarker mining have indicated that *Pseudolabrys* is highly sensitive to different fertilisation regimes and may serve as a key indicator for predicting crop yield fluctuations and the soil phosphorus activation capacity [20]. *Pseudolabrys* has been positively associated with wheat yield. Under controlled-release urea and fulvic acid treatments, wheat yield increased by 22.1%, and the relative abundance of *Pseudolabrys* increased [21]. In the present study, *Pseudolabrys* showed a positive association with wheat yield (Figure 5A) and was related to the fertilisation rate (Figure 4B).

**Figure 6:**
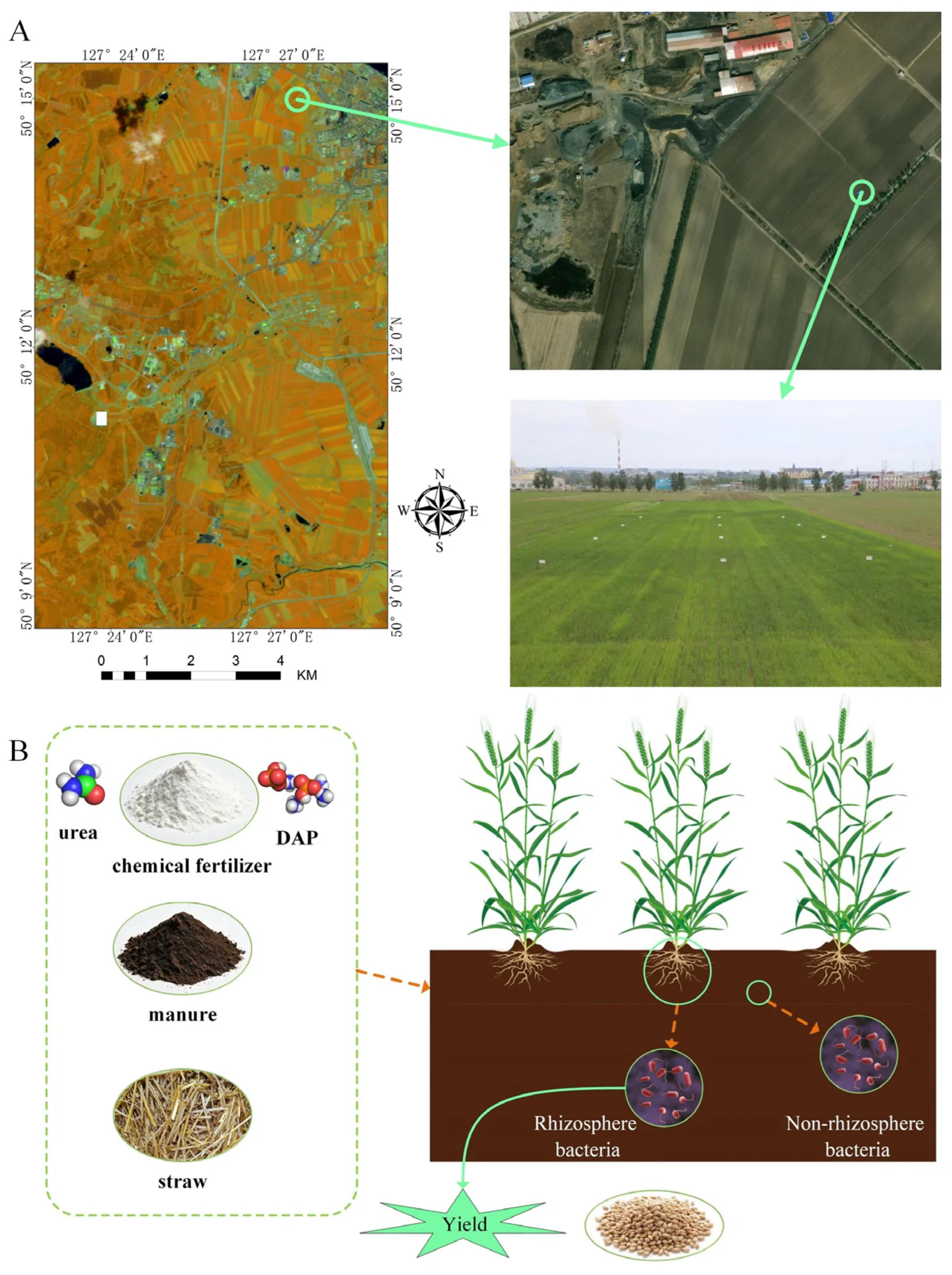
Location of soil sampling sites and experimental design. (**A**) Geographic location of the experimental field. (**B**) Experimental design showing the collection and comparative analysis of rhizosphere and non-rhizosphere soil microbial samples and wheat and soybean yield under long-term fertilisation treatments.

In the present study, *Gemmatirosa* was positively associated with soybean yield and related to the chemical fertiliser application rate (Figure 5B and Figure 6A). To our knowledge, no previous studies have reported a positive association between *Gemmatirosa* and soybean yield, making this relationship worthy of further investigation. Under saline–alkaline conditions, *Gemmatirosa* in the rhizosphere soil of salt-tolerant soybean cultivars has been significantly correlated with malondialdehyde [22]. A survey of soybean-growing soils on South African farms reported that *Gemmatirosa* was widely distributed; however, because it could not be cultured, its effects on plant growth were not clearly determined [23]. A long-term fertilisation experiment on calcareous soil showed that fertilisation reduced the relative abundance of *Gemmatirosa* in soil [24].

### 3.3. Exploratory Molecular Docking of a Rhizosphere Bacterial Protein

To move past the limitations of conventional omics-based inference, this study investigated the functional mechanisms of dominant bacteria at the level of molecular binding. *Lysobacter* remained highly abundant under all treatments and was positively correlated with soybean yield (Figure 5A). This bacterium is beneficial to soybean and wheat growth, improves the microbial environment around plant roots, and effectively suppresses soybean and wheat root rot, which is mainly caused by *Fusarium* spp. [25,26]. *Lysobacter* can also enhance stress resistance in soybean and wheat [27,28] and plays an important role in nutrient cycling [29].

Molecular docking simulations confirmed that PstS of *Lysobacter* formed a compact three-dimensional conformation with the phosphite substrate and exhibited a stable binding energy of −4.93 kcal/mol. This atom-scale evidence of a molecular interaction was consistent with the high abundance of the ABC transporter pathway in the metagenomic data (Supplementary Figure S3), indicating how microorganisms under combined organic–mineral fertilisation may use surface functional proteins to efficiently capture and transport small-molecule nutrients, thereby promoting growth.

## 4. Materials and Methods

### 4.1. Experimental Design and Microecological Sample Collection

This study focused on a long-term wheat–soybean rotation field experiment (Figure 6A) at the Heihe Branch of the Heilongjiang Academy of Agricultural Sciences (127.46° E, 50.26° N; elevation, 160 m). A satellite remote-sensing map of the experimental site was generated using ArcGIS (ESRI ArcMap™, version 10.8).

The experiment was conducted continuously for 46 years from 1979 to 2025. The following 12 fertilisation treatments were established in individual 200 m^2^ plots (20 m × 10 m), with one field plot per treatment and three within-plot spatial soil samples for microbiome profiling: unfertilised control (CK); wheat/soybean straw return alone (S); organic manure alone (M); low-rate chemical fertiliser (NP); medium-rate chemical fertiliser (2NP); high-rate chemical fertiliser (4NP); straw combined with different chemical fertiliser rates (S + NP, S + 2NP, and S + 4NP); and organic manure combined with different chemical fertiliser rates (M + NP, M + 2NP, and M + 4NP). The corresponding nominal mineral inputs were 37.5, 75, or 150 kg N ha^−1^ and the same numerical rates in kg P_2_O_5_ ha^−1^; no mineral K_2_O was applied. Urea (46% N) and diammonium phosphate (18% N and 46% P_2_O_5_) supplied the mineral nutrients; the N supplied by diammonium phosphate must be included in the stated total N rate. In S treatments, 3000 kg ha^−1^ of wheat straw on an air-dry basis was returned. In M treatments, 15,000 kg ha^−1^ of farmyard manure was applied once every three years. Rhizosphere and non-rhizosphere soils were sampled on 3 June 2025 during the wheat phase at three spatially separated positions within each treatment plot. At each position, five soil subsamples were combined to form a composite sample; these samples were within-plot subsamples, not independent field-plot replicates. Rhizosphere soil was defined as soil tightly adhered to the root surface, within 1–2 mm, and non-rhizosphere soil was collected from the 0–15 cm layer between crop rows where no roots were present. Wheat root morphology and root hair length were observed under the different treatments using scanning electron microscopy.

### 4.2. Crop Yield Analysis

The grain and straw yields of wheat and soybean under each fertilisation treatment were recorded from 1979 to 2025, and all yield data were standardised to kg ha^−1^. During data preprocessing, records with a value of 0 were replaced with NA and excluded to ensure statistical rigor. The long-term distributions of the yield variables were visualised across the 12 treatments as boxplots using the ‘ggplot2’ package in R (version 4.5.3), and the original treatment comparisons used one-way analysis of variance, which does not account for year, fertilisation × year interactions, or repeated observations of the same field plots.

### 4.3. Metagenomic High-Throughput Sequencing and Functional Annotation

Total genomic DNA was extracted from the collected rhizosphere and non-rhizosphere soil samples. Paired-end metagenomic sequencing was performed on the Illumina platform. After quality control, adapter-containing reads, reads with more than 10% ambiguous bases, and reads for which at least half of the bases had quality scores of 10 or lower were removed; reads assigned to the host were then excluded. Clean reads were assembled with MEGAHIT, and contigs containing at least 500 bp were retained. Open reading frames were predicted with Prodigal; predicted genes were dereplicated with CD-HIT and reads were mapped to the resulting gene catalogue with Bowtie2 to estimate gene abundance as transcripts per million (TPM). For taxonomic annotation, predicted proteins were compared with NCBI NR by DIAMOND (E-value threshold 1 × 10^−5^), and ambiguous hits were assigned taxonomically using the lowest common ancestor procedure in MEGAN. Functional annotations were generated by DIAMOND searches against Kyoto Encyclopedia of Genes and Genomes (KEGG), with KEGG Orthology (KO) abundances identified from the annotated gene-abundance profiles.

### 4.4. Diversity of Rhizosphere and Non-Rhizosphere Soil Bacterial Communities

The Shannon diversity index was calculated from rhizosphere and non-rhizosphere metagenomic taxonomic data and visualised using the ‘vegan’ and ‘ggplot2’ packages in R (version 4.5.3). Differences among treatments were determined using the Kruskal–Wallis test. Shannon indices were compared between paired rhizosphere and non-rhizosphere samples, and a paired *t*-test was used to assess the significance of the effect of the rhizosphere on bacterial diversity.

The functional community structure was evaluated using principal coordinate analysis (PCoA), comparing KO functional gene matrices from rhizosphere and non-rhizosphere samples based on Bray–Curtis distances. Two separate one-factor permutational analyses of variance (PERMANOVAs) were originally performed, one on rhizosphere KO profiles and one on non-rhizosphere KO profiles, with fertilisation treatment as the sole factor in each model (Table 1). Each compartment contained 36 profiles (12 treatments × three within-plot composites).

### 4.5. Relative Abundance of Dominant Microorganisms at the Genus Level

Based on the metagenomic sequencing data, the relative abundance of the top 20 dominant microbial genera was calculated for each treatment. The community composition was visualised using stacked bar plots, and means ± standard deviations were calculated to compare the effects of fertilisation treatments on dominant microbial groups.

### 4.6. Molecular Docking of a Bacterial Protein with a Small Molecule

The structure of the phosphate ABC transporter substrate-binding protein (PstS; WP_051884339.1) from the most abundant bacterium, *Lysobacter*, was downloaded from the National Center for Biotechnology Information (Bethesda, MD, USA) to investigate the molecular mechanism by which this core bacterial genus participates in nutrient transformation. Molecular docking with phosphite (CID_107908) was performed using AlphaFold 3 (https://alphafoldserver.com). The binding energy of the resulting complex was calculated using PRODIGY (https://wenmr.science.uu.nl/prodigy, 3 August 2026), and protein–small-molecule docking was visualised using PyMOL software (v3.1, www.pymol.org).

### 4.7. Differential Rhizosphere Taxa and KO Functional Gene Analysis

Based on rhizosphere metagenomic taxonomic data, linear discriminant analysis (LDA) effect size (LEfSe) was used to identify rhizosphere-specific biomarkers for each treatment from the phylum to genus levels, and LDA scores (>3.0) were calculated for differentially abundant taxa.

STAMP software (v2.1.3) was used to conduct pairwise differential analysis of the genus-level rhizosphere microbial composition among the 12 treatments. Genera with significantly different abundances between treatment pairs were identified to characterise key microbial groups enriched or depleted by the rhizosphere effect.

Based on TPM-normalised KO functional gene data, the mean relative abundances of the top 50 functional genes and enriched pathways for each treatment were calculated. A heatmap was used to visualise differences in functional gene (pathway) abundance among fertilisation treatments and to identify key functional genes (pathways) enriched or downregulated under high-rate chemical fertiliser and organic manure treatments.

### 4.8. Correlation Analysis Between Dominant Rhizosphere Genera and Crop Yield

Pearson correlations were originally explored between the relative abundances of the top 20 genera in wheat rhizosphere samples collected on 3 June 2025 and four yield variables: wheat grain, wheat straw, soybean grain, and soybean straw yields. For each treatment, these yield variables were means of the available harvest records from the 46-year field experiment, rather than yields from the microbiome sampling year alone. These exploratory correlations compare a single 2025 microbiome snapshot with historical treatment-level yield summaries.

### 4.9. Structural Equation Modelling

The originally reported structural equation model combined 36 within-plot soil profiles from 12 treatment plots with historical treatment-level yield summaries and was fitted using the ‘piecewiseSEM’ package in R (version 4.5.3) to quantify the pathways and effect sizes through which fertilisation treatments affected crop yield (wheat/soybean grain and straw) by regulating soil physicochemical properties (pH, AP, and AK) and microbial diversity (Shannon index of the rhizosphere metagenome).

## 5. Conclusions and Perspectives

Sustaining high productivity in the wheat–soybean rotation system across generations depends on close coupling between soil physicochemical properties and rhizosphere biological characteristics. Long-term organic manure application alleviated the physicochemical stress caused by chemical fertiliser alone and optimised the assembly of core rhizosphere microbial groups with potential functions in nitrogen fixation and nutrient activation. Future studies should expand microecological modelling by integrating long-term field-scale big-data networks with computational structural biology of multi-target bacterial proteins to comprehensively elucidate the molecular metabolic networks underlying microbe–plant interactions and communication in the rhizosphere.

## Figures and Tables

**Table 1:** Separate treatment-only PERMANOVAs of KO functional profiles for rhizosphere and non-rhizosphere soils.

Soil	Source	Df	SumOfSqs	R^2^	F	*p*_Value
Rhizosphere	Treatment	11	0.3784	0.7578	6.83	0.001
Rhizosphere	Residual	24	0.1209	0.2422		
Rhizosphere	Total	35	0.4993	1		
Non-rhizosphere	Treatment	11	0.3692	0.8227	10.12	0.001
Non-rhizosphere	Residual	24	0.0796	0.1773		
Non-rhizosphere	Total	35	0.4488	1		

## Data Availability

Please contact the author to request the data.

## Supplementary Materials

The following supporting information can be downloaded at: https://www.mdpi.com/article/10.3390/plants15193034/s1, Supplementary Figure S1. Biomarker discovery via LEfSe analysis. Supplementary Figure S2. STAMP analysis of differential genera. Supplementary Figure S3. Heatmap of the top 50 enriched KO pathways.
