# Supplementary material for: Long-Term Fertilisation Regimes, Crop Yield and Rhizosphere Microecology in a Wheat–Soybean Rotation System
Source: Plants (Basel). 2026 Oct 4;15(19):3034. doi: 10.3390/plants15193034 (PMC13667143; doi:10.3390/plants15193034)
Supplement: Supplementary file 1 [file plants-15-03034-s001.zip › plants-4575252-supplementary.pdf]

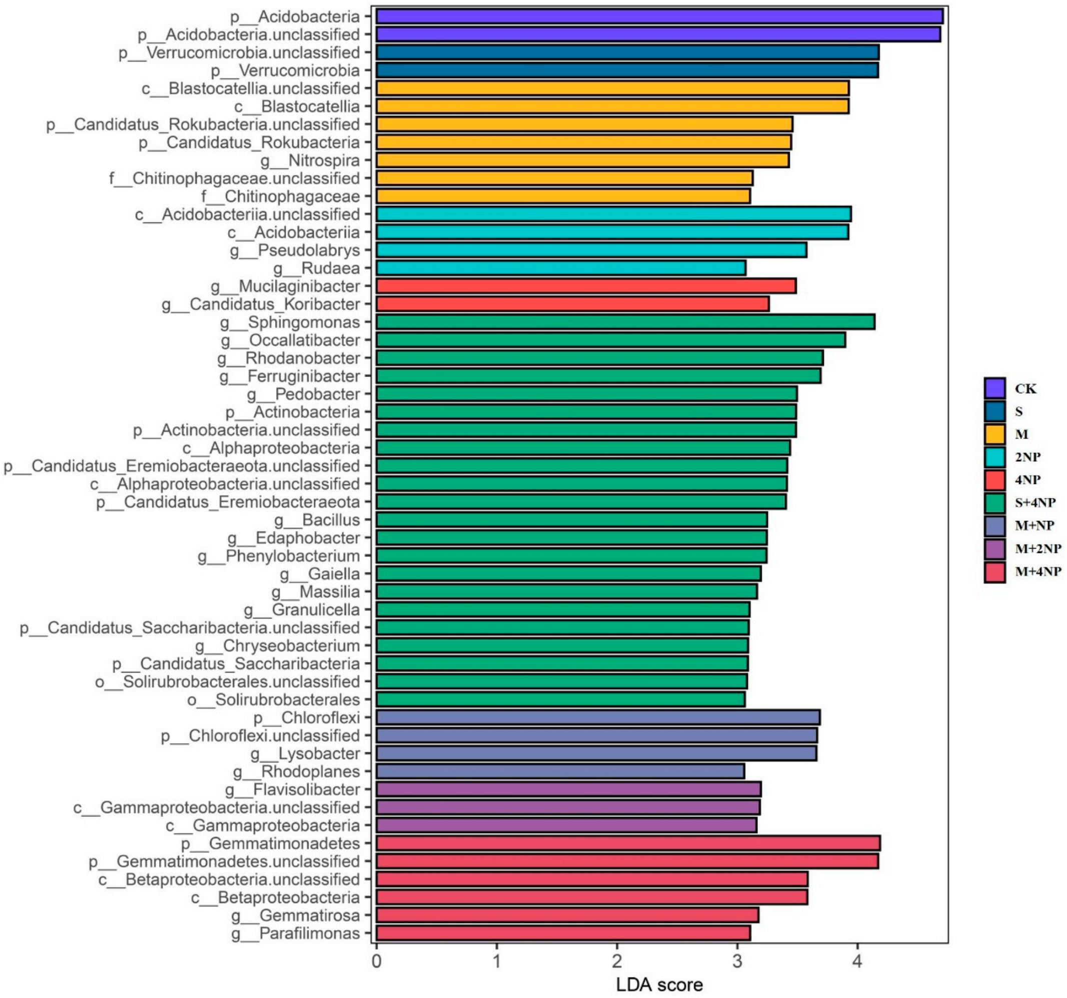

Supplementary Figure S1. Biomarker discovery via LEfSe analysis. The bar plot shows linear discriminant analysis (LDA) scores for differential indicator taxa at different taxonomic levels (threshold > 3.0).

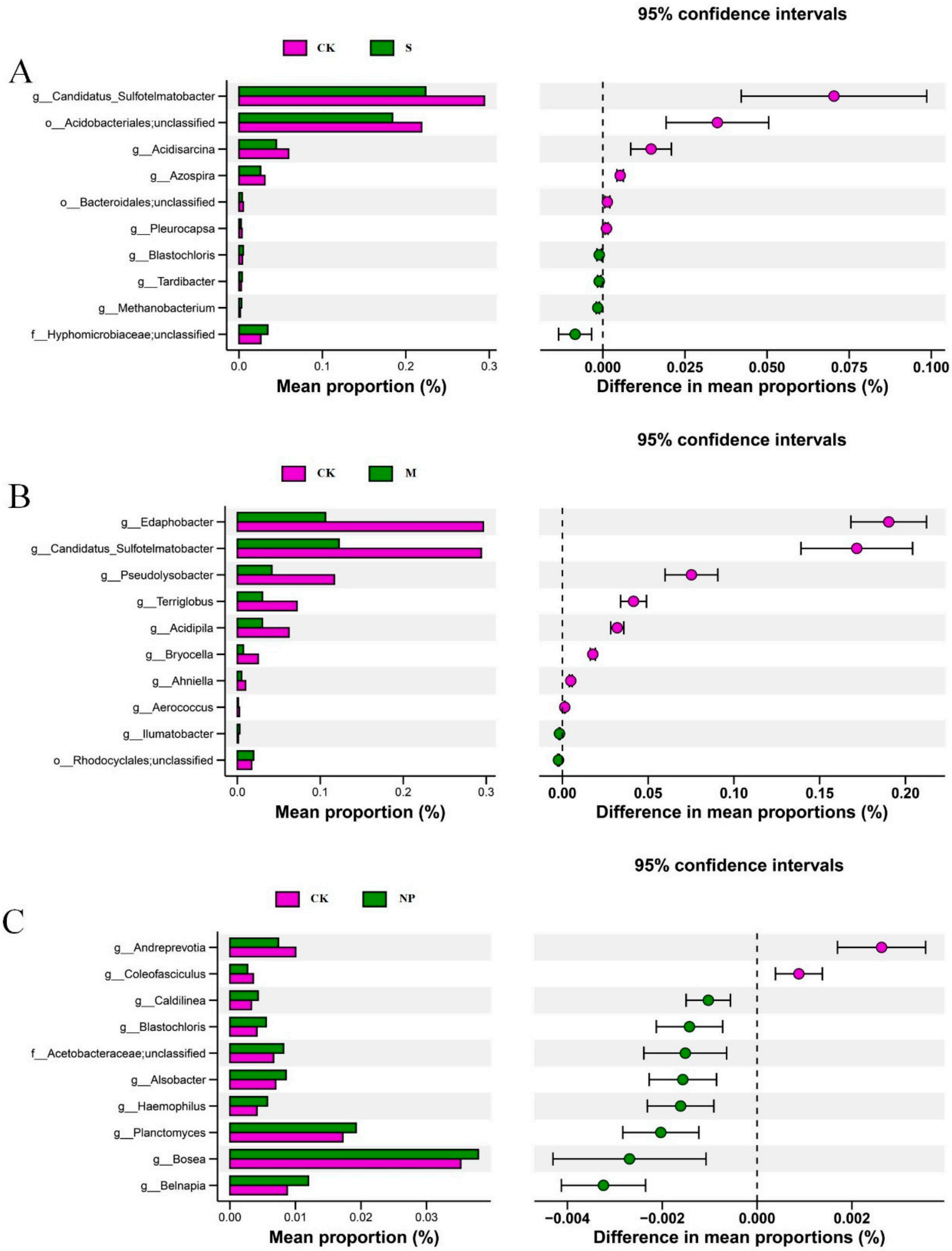

Supplementary Figure S2. STAMP analysis of differential genera. Significant differences in the relative abundance of dominant rhizosphere genera and their confidence intervals are shown for different fertilisation treatment comparisons (A, CK vs. S; B, CK vs. M; C, CK vs. NP).

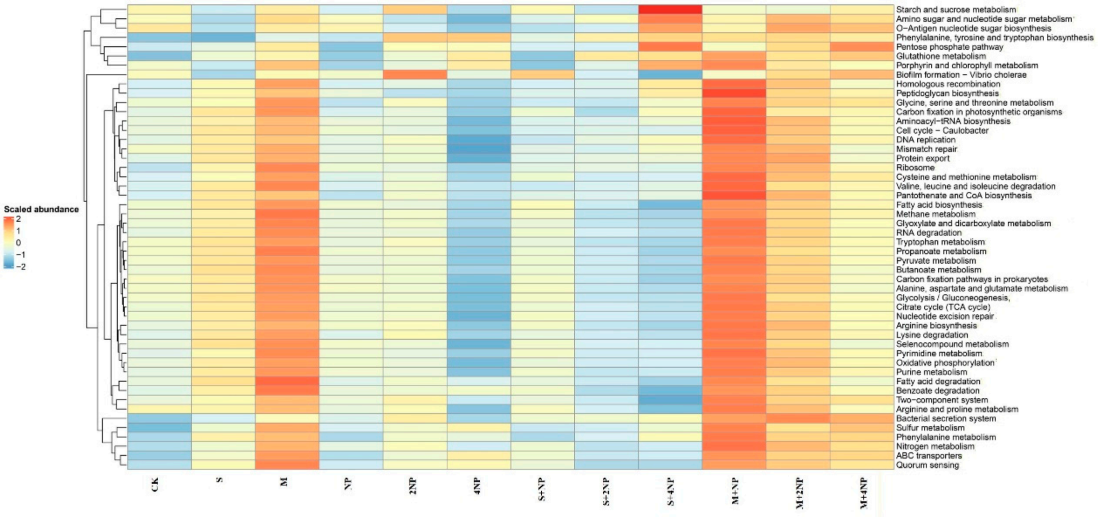

Supplementary Figure S3. Heatmap of the top 50 enriched KO pathways. Clustered heatmap of the abundance (TPM) of the top 50 KEGG Orthology (KO)-enriched metabolic pathways in rhizosphere microbial communities under different fertilisation treatments.
